# Gut Microbiome Changes After Neoadjuvant Chemotherapy and Surgery in Patients with Gastric Cancer

**DOI:** 10.3390/cancers16234074

**Published:** 2024-12-05

**Authors:** Kristina Žukauskaitė, Bernardas Baušys, Angela Horvath, Rasa Sabaliauskaitė, Agnė Šeštokaitė, Agata Mlynska, Sonata Jarmalaitė, Vanessa Stadlbauer, Rimantas Baušys, Augustinas Baušys

**Affiliations:** 1 Institute of Biosciences, Life Science Center, Vilnius University, 01513 Vilnius, Lithuania; kristina.zukauskaite@medunigraz.at (K.Ž.);; 2 Division for Gastroenterology and Hepatology, Department of Internal Medicine, Medical University of Graz, 8010 Graz, Austria; 3 Institute of Clinical Medicine, Faculty of Medicine, Vilnius University, 03101 Vilnius, Lithuania; 4 Division of Translational Precision Medicine, Center for Biomarker Research in Medicine (CBmed GmbH), 8010 Graz, Austria; 5 Laboratory of Genetic Diagnostics, National Cancer Institute, 08406 Vilnius, Lithuania; 6 Laboratory of Immunology, National Cancer Institute, 08406 Vilnius, Lithuania; 7 Department of Chemistry and Bioengineering, Vilnius Gediminas Technical University, 10223 Vilnius, Lithuania; 8 Department of General and Abdominal Surgery and Oncology, National Cancer Institute, 08406 Vilnius, Lithuania; 9 Laboratory of Experimental Surgery and Oncology, Translational Health Research Institute, Faculty of Medicine, 03101 Vilnius, Lithuania

**Keywords:** gastric cancer, gastrectomy, neoadjuvant chemotherapy, oralization, gut microbiome, dysbiosis, radical surgery, FLOT

## Abstract

Background/Objectives: Neoadjuvant chemotherapy (NAC) followed by radical gastrectomy is the current standard approach for locally advanced gastric cancer (GC) in the West. Both NAC and gastrectomy can significantly influence the gut microbiome, potentially leading to clinically significant changes. However, no longitudinal studies to date support this hypothesis. This study investigates gut microbiome changes throughout GC treatment, including NAC and gastrectomy. Methods: This longitudinal observational study included GC patients undergoing NAC followed by gastrectomy. Fecal microbiome composition, intestinal inflammation (fecal calprotectin), and gut permeability (LBP, sCD14) markers were investigated at baseline, after NAC, and after gastrectomy. Results: A total of 38 patients were included in the study. The results showed that NAC did not affect the gut microbiome composition at the phylum level. In contrast, radical gastrectomy led to an increased abundance of Bacteroidetes and Proteobacteria and a decreased abundance of Firmicutes and Actinobacteria. Furthermore, NAC alone did not impact alpha or beta diversity, while a combination of NAC and gastrectomy significantly influenced both. After gastrectomy, the gut microbiome composition analysis also revealed enrichment of oralization-associated bacterial species such as *Escherichia-Shigella*, *Streptococcus equinus*, uncultured *Streptococcus* species, and species from the Enterobacteriaceae family. Intestinal inflammation and gut permeability markers did not significantly change throughout the treatment. Conclusions: The radical treatment of advanced GC with NAC and radical surgery has long-term effects on the gut microbiome, characterized by gut microbiome oralization. These sustained alterations primarily stem from the radical gastrectomy rather than the NAC. Since previous studies have linked oralization-associated dysbiosis to various gastrointestinal symptoms, this study highlights the gut microbiome as a potential therapeutic target to enhance the quality of life in long-term survivors following gastrectomy.

## Simple Summary

Neoadjuvant chemotherapy (NAC) followed by radical gastrectomy is the standard approach for locally advanced gastric cancer (GC) in the West. This study investigated microbiome changes throughout GC treatment, including NAC and gastrectomy. A longitudinal observational design was employed, analyzing gut microbiome composition, fecal calprotectin, and gut permeability markers (LBP, sCD14) at baseline, post-NAC, and post-gastrectomy in 38 patients. Results indicated that NAC did not alter gut microbiome composition at the phylum level, whereas gastrectomy increased Bacteroidetes and Proteobacteria and decreased Firmicutes and Actinobacteria. NAC alone did not affect alpha or beta diversity; however, combining NAC with gastrectomy led to significant diversity changes. Post-gastrectomy microbiome analysis revealed an enrichment of oralization-associated bacteria, including *Escherichia-Shigella*, *Streptococcus equinus*, and members of the Enterobacteriaceae family. These findings suggest that gut microbiome alterations in GC treatment are primarily driven by gastrectomy rather than NAC, resulting in long-term oralization-associated shifts.

## 1. Introduction

Gastric cancer (GC) ranks as the fifth most common malignancy and the fourth leading cause of cancer mortality worldwide, with over 1 million new cases and approximately 769,000 deaths annually [1]. The current standard curative approach for locally advanced GC involves neoadjuvant chemotherapy (NAC) followed by radical surgery. Despite advancements in treatment strategies over recent decades, managing GC remains challenging due to poor survival rates [2], high incidence of treatment-related complications [3], and impaired quality of life in long-term survivors [4,5]. Gastrectomy leads to significant anatomical and physiological alterations in the gastrointestinal tract, including increased gastric pH resulting from reduced gastric acid secretion [6,7]. Studies on proton-pump inhibitor (PPI) users have suggested that gastric acid suppression significantly affects the distal gastrointestinal microbiome [8,9,10]. Our previous proof-of-concept study demonstrated that the loss of the gastric barrier post-gastrectomy results in gut microbiome oralization, characterized by an increased abundance of Escherichia-Shigella, Enterococcus, Streptococcus, and other typical oral cavity bacteria (*Veillonella*, *Oribacterium*, *Mogibacterium*) [6]. This gastrectomy-induced dysbiosis is clinically significant, as gut microbiome oralization is associated with intestinal inflammation, characterized by increased fecal calprotectin and gastrointestinal symptoms [6]. Furthermore, chemotherapy is known to induce microbial dysbiosis in the gut [11,12,13], potentially contributing to gastrointestinal adverse events such as diarrhea [14,15]. Notably, the extent of gut microbiome alterations varies with different chemotherapy regimens [11]. These insights suggest that targeting the gut microbiome could be a promising therapeutic approach to enhance quality of life and overall health in cancer patients undergoing treatment and long-term survivors. However, there is a lack of studies investigating the impact of modern neoadjuvant chemotherapy regimens, such as FLOT (5-fluorouracil, leucovorin, oxaliplatin, docetaxel) on gut microbiome composition in GC patients. Additionally, evidence for gastrectomy-induced dysbiosis has been limited by the cross-sectional design of previous studies. Therefore, this longitudinal observational study aims to elucidate the changes in the gut microbiome throughout GC treatment and to delineate the specific effects of NAC and radical surgery, along with their impact on intestinal inflammation and gut permeability.

## 2. Materials and Methods

### 2.1. Study Design and Ethics

This longitudinal observational study is a side-study of a previous RCT investigating prehabilitation before gastrectomy registered in the https://clinicaltrials.gov/ (NCT04223401) registry. The study protocol [16] and results [3] has been published previously. The study protocols and all subsequent amendments received approval from the Vilnius Regional Bioethics Committee (2020/1-1185-675). The study was conducted following the ethical standards of the Helsinki Declaration of 2013. All patients provided written informed consent before participation.

### 2.2. Study Participants

Patients aged 18 years or older, scheduled to undergo elective GC radical surgery after NAC as determined by the decision of a multidisciplinary team at the National Cancer Institute, Vilnius, Lithuania, were eligible for this side-study. The exclusion criteria matched those used in the RCT: (a) surgery due to GC recurrence, (b) patient’s conditions did not allow surgery to be postponed for at least 4 weeks, and (c) inability to participate in the prehabilitation program due to the patient’s physical or mental condition. Additional exclusion criteria specific to this side-study included the following: (d) chemotherapy or radiotherapy within 12 months before inclusion; (e) use of antibiotics, pro-, pre-, or synbiotics within 1 month of inclusion; (f) history of any major gastrointestinal tract resections; and (g) current non-gastric malignancies.

### 2.3. Blood and Stool Sample Collection

Blood and fresh stool samples were collected at baseline (BL), before the surgery within a week of NAC (post-NAC), and 12 months after the start of the treatment, ensuring a minimum of 6 months post-surgery (post-SX), and immediately stored at −80 °C until the DNA extraction and other experiments.

### 2.4. 16S rRNA Gene Sequencing

According to the manufacturer’s recommendations, DNA from frozen stool samples was extracted using the SphaeraMag^®^ Genomic DNA Fecal Purification Kit (Procomcure Biotech, Austria). The samples were processed one by one in tubes to reduce the chance of cross-contamination. Library preparations and *16S rRNA* gene sequencing were conducted using the Illumina protocol. Hypervariable regions V1–V2 were amplified (primers: 27F-AGAGTTTGATCCTGGCTCAG; R357-CTGCTGCCTYCCGTA) and sequenced using an Illumina NextSeq2000 instrument according to the application note. Raw sequencing data are made publicly available in the National Centre for Biotechnology Information (NCBI) sequence read archive (SRA) at https://www.ncbi.nlm.nih.gov/sra (accessed on 14 September 2024) (SRA data accession No. PRJNA1124058).

### 2.5. Processing of the Sequencing Data

Raw next-generation sequencing data were demultiplexed using BaseSpace Sequence Hub with the application BaseSpace DRAGEN Analysis (v1.3.0) or BCL Convert (v2.4.0). Raw sequencing data were processed using QIIME 2 tools on a local Galaxy instance (https://www.medunigraz.at/ (accessed on 14 September 2024)) [17], and quality was checked with FastQC and MultiQC. Based on the quality reports to ensure the integrity of the sequencing data and based on the quality report, forward and reverse read sequences were truncated at 280 and 250 bases, respectively. Denoising was undertaken with DADA2, made available through the QIIME2 tool [18,19]. After processing, filtering, and rarefying the sequencing data, 13,476,299 sequencing reads remained. On average, each sample had 132,120 reads available for further analysis, with a minimum of 58,842 reads and a maximum of 277,248 reads. Taxonomy was assigned based on the Silva 132 database release at 99% operational taxonomic unit level with a naïve Bayes classifier. A phylogenetic tree was built by creating a sequence alignment using MAFFT. The resulting masked alignment was used to infer a phylogenetic tree and then root it as its midpoint using FastTree.

The resulting count table, classification, and rooted tree were imported into the R-based CBmed Microbiome Analysis Platform using the qza_to_phyloseq() function from the qiime2R package [20]. Cyanobacteria were removed from the resulting phyloseq object as potential contaminants.

### 2.6. Measurement of Inflammation and Gut Permeability Biomarkers

The enzyme-linked immunosorbent assay (ELISA) was used to measure fecal calprotectin (Hycult Biotech, Uden, The Netherlands), serum lipopolysaccharide-binding protein (LBP; Nordic Biosite, 1301 Copenhagen, Denmark), and soluble CD14 (sCD14; Diaclone, Besancon, France).

### 2.7. Statistical Analysis

Alpha diversity analysis was quantified by the Richness, Shannon, Inverse Simpson, Evenness, and Phylogenetic Diversity indices. Beta diversity was examined by Principal Coordinates Analysis (PCoA) based on the unique fraction metric (*unifrac*), weighted unifrac (*wunifrac*), and the *Bray–Curtis* and *Jaccard* dissimilarity matrices, and results were evaluated using Permutational Multivariate Analysis of Variance (PERMANOVA) using R (R Core Team, 2023, version 4.3.0) through the RStudio interface and the *vegan* package [21]. The PERMANOVA results were confirmed by redundancy analysis (RDA), which was conducted using the *vegan* package. Subsequently, Linear discriminant analysis Effect Size (LEfSe) analysis was performed to identify features that exhibit differential abundance between different treatment groups and to determine their effect sizes using the *microbiomeMarker* package [22]. A Linear Discriminant Analysis (LDA) cutoff value of 4 was applied uniformly to treatment groups, enabling the identification of substantial differences in abundance with increased precision. Subsequently, a linear model was employed to ascertain the statistical significance of the obtained results using the lme4 package [23]. Figures were created using the *ggplotify* package [24].

## 3. Results

### 3.1. Patient Characteristics

Between 13 April 2021 and 22 September 2022, 38 GC patients were included in the study. Table 1 presents the baseline and treatment characteristics of the study participants. Patients diagnosed with distant metastases exhibited positive peritoneal cytology and no other distant metastases. After enrolment in the study, all patients underwent NAC followed by radical surgery.

### 3.2. NAC and Gastrectomy Impact on Alpha and Beta Diversity

**Figure 1:**
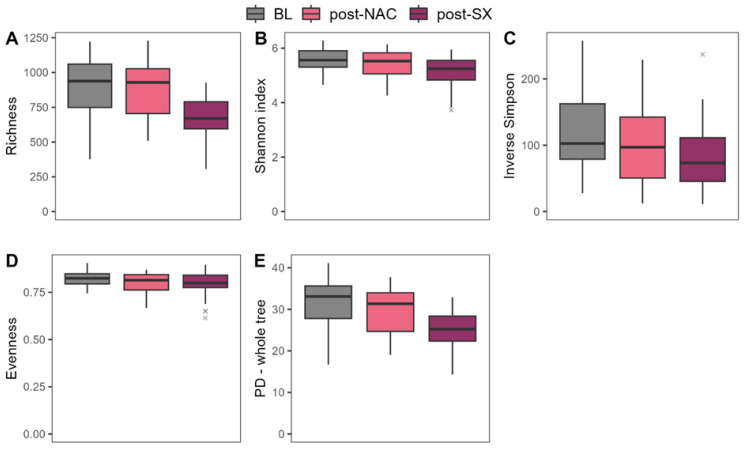
Changes in alpha-diversity parameters in patients undergoing advanced gastric cancer treatment; outliers are marked in grey; Alpha-diversity parameters: (**A**) Richness, (**B**) Shannon index, (**C**) Inverse Simpson index, (**D**) Evenness, (**E**) Phylogenetic Diversity; BL–baseline, NAC–neoadjuvant chemotherapy, SX–surgery.

Our study revealed that NAC did not affect alpha and beta diversity, whereas radical surgery affected both. Alpha diversity, assessed by the Richness (*p* < 0.0001), Shannon (*p* = 0.014), and Phylogenetic Diversity indices (*p* < 0.0001), decreased significantly after radical surgery, compared to NAC. Additionally, the Inverse Simpson and Evenness indices showed a tendency to decrease but did not reach statistical significance (both *p* > 0.050) (Figure 1, Table S1).

**Figure 2:**
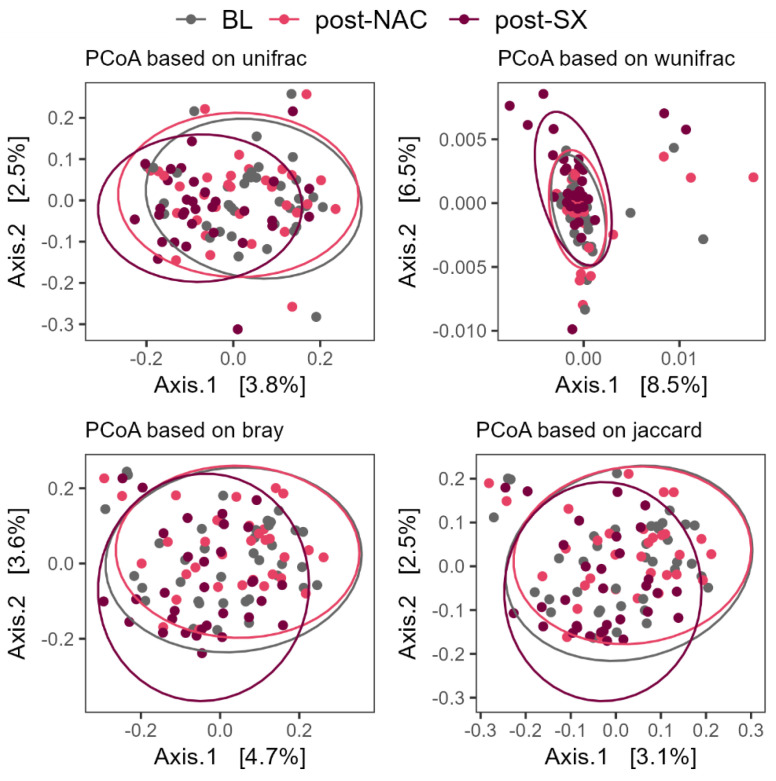
Changes in beta-diversity parameters in patients undergoing chemotherapy and surgery combined with chemotherapy; BL–baseline, NAC–neoadjuvant chemotherapy, SX–surgery.

Beta diversity analysis, using principal coordinates analysis (PCoA) based on the unique fraction metric (*unifrac*), weighted unifrac (*wunifrac*), and the *Bray–Curtis*, and *Jaccard* dissimilarities, showed a significant difference between post-surgery and post-NAC timepoints (all *p* = 0.001) (Figure 2, Table S2). Redundancy analysis confirmed the profound impact of radical surgery on the gut microbiome changes (*p* = 0.014).

### 3.3. Microbiome Composition Changes Throughout the Treatment of GC

**Figure 3:**
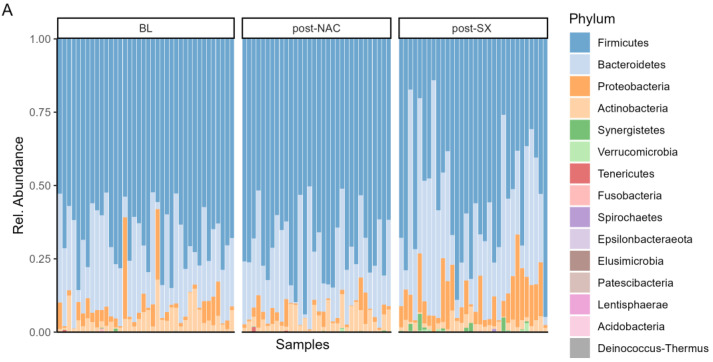

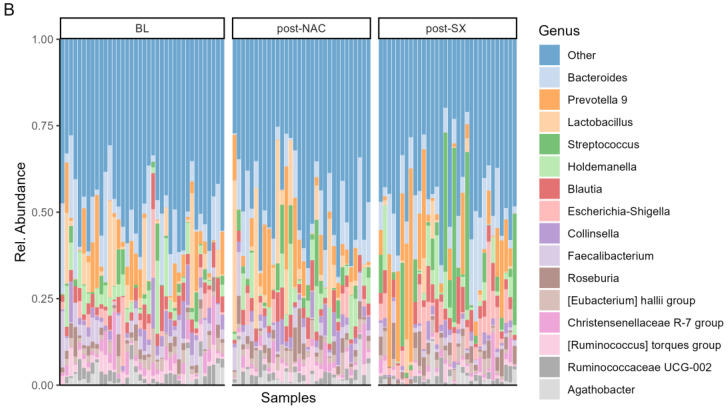
Bacterial composition of the gut microbiome at the (**A**) phylum and (**B**) genus level at different treatment timepoints; BL–baseline, NAC–neoadjuvant chemotherapy, SX–surgery (radical gastrectomy).

The study showed that the gut microbiome composition changed significantly throughout the treatment of GC. At the phylum level, NAC treatment did not alter the microbiome composition. In contrast, radical surgery led to an increased abundance of Bacteroidetes (*p* = 0.004) and Proteobacteria (*p* < 0.0001) and a decreased abundance of Firmicutes (*p* < 0.0001) and Actinobacteria (*p* = 0.001) compared to NAC (Figure 3A, Table S3).

At the genus level, NAC treatment resulted in a significant decrease in the *Christensenellaceae R-7* group (*p* = 0.041) compared to BL samples. In contrast, radical surgery led to an increase in *Prevotella 9* (*p* = 0.022), *Streptococcus* (*p* = 0.010), and *Escherichia-Shigella* (*p* = 0.001). This combination also caused a decrease in *Lactobacillus* (*p* = 0.008), *Collinsella* (*p* = 0.005), *Faecalibacterium* (*p* = 0.030), and the *Ruminococcus torques* group (*p* = 0.006) (Figure 3B, Table S4).

### 3.4. The Microbiome Composition After Surgery Is Enriched with Oralization-Associated Bacteria

**Figure 4:**
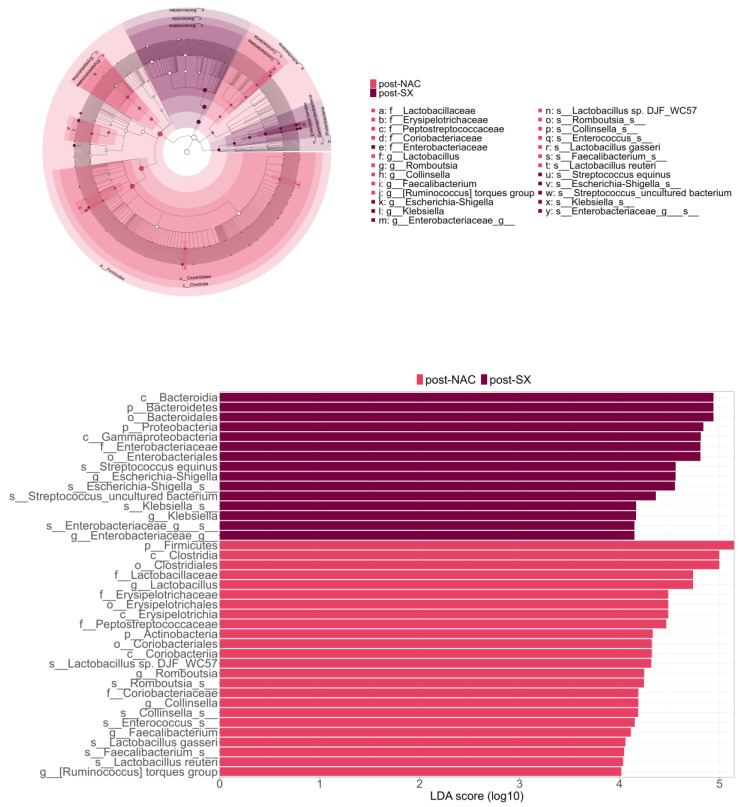
The primary microbiome differences between post-surgery (post-SX) and post-neoadjuvant chemotherapy (post-NAC) treatment in patients with advanced gastric cancer.

LEfSe analysis supported these findings, demonstrating that, post-surgery, patients exhibited a more enriched microbiome characterized by a higher abundance of *Escherichia-Shigella* species, *Streptococcus equinus*, uncultured *Streptococcus* species, and members of the Enterobacteriaceae family (Figure 4).

**Figure 5:**
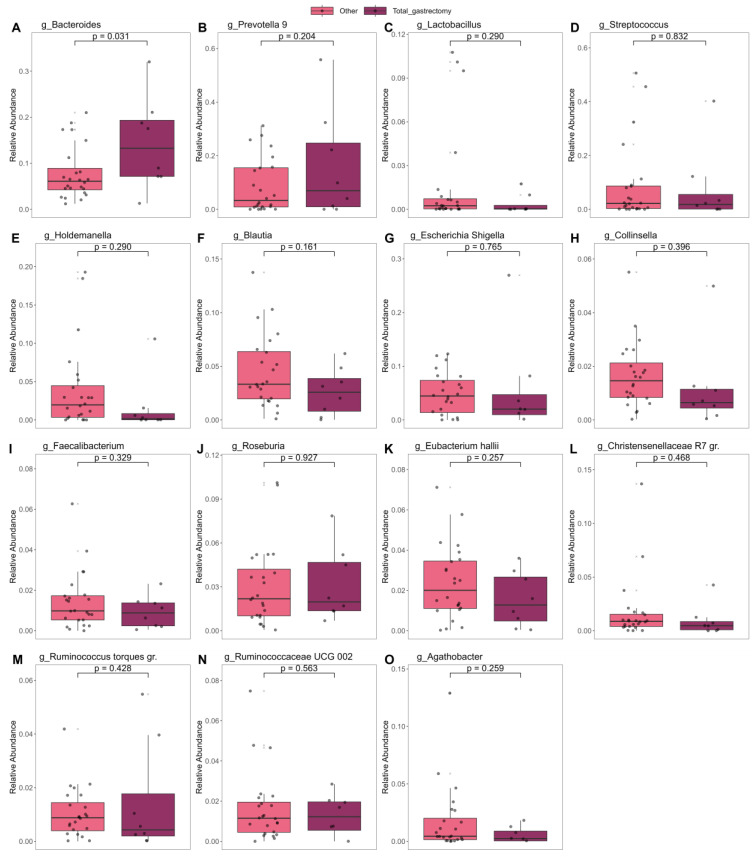
Comparative analysis of the relative abundance of various genera in patients undergoing total or subtotal gastrectomy.

Additionally, ANCOM validated these results by demonstrating that post-SX patients had higher levels of *Escherichia-Shigella* and lower levels of *Faecalibacterium* and the *Ruminococcus torques* group. Linear models showed that the type of surgery, total or subtotal gastrectomy, had no impact on the gut microbiome composition except for the higher levels of *Bacteroides* in patients who received a total gastrectomy (Figure 5, Table S5).

### 3.5. Fecal and Blood Biomarkers Throughout the Treatment of GC

**Figure 6:**
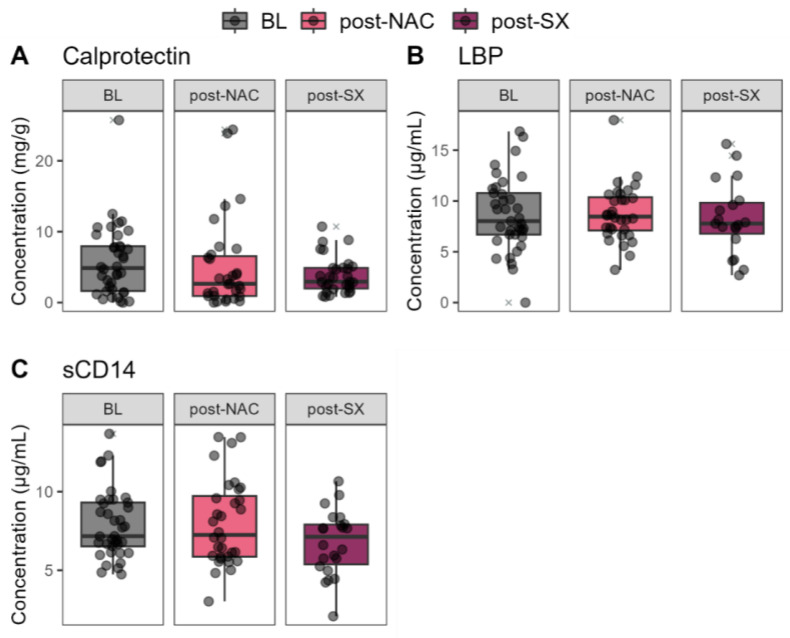
Blood parameters in gastric cancer patients post-surgery (post-SX) and post-neoadjuvant chemotherapy (post-NAC) treatment, compared to the baseline (BL).

Fecal calprotectin, lipopolysaccharide-binding protein (LBP), and soluble Cluster Differentiation 14 (sCD14) biomarkers from the blood serum did not significantly change throughout the treatment (Figure 6).

## 4. Discussion

This study aimed to investigate gut microbiome changes throughout GC treatment with NAC and radical surgery and to explore the effect of treatment. The results indicate significant changes in the gut microbiome during treatment, driven primarily by radical surgery rather than NAC. NAC alone did not impact the gut microbiome composition at the phylum level. In contrast, radical surgery led to an increased abundance of Bacteroidetes and Proteobacteria phylum and a decreased abundance of Firmicutes and Actinobacteria phylum. Furthermore, NAC did not affect alpha or beta diversity, whereas radical surgery impacted both. The microbiome composition after radical surgery was enriched with oralization-associated bacteria, as LEfSe analysis showed an increased abundance of *Escherichia-Shigella*, *Streptococcus equinus*, and uncultured bacterium *Streptococcus* species, and species from Enterobacteriaceae family. ANCOM validated these results by demonstrating higher levels of *Escherichia-Shigella* and lower levels of *Faecalibacterium* genus and the *Ruminococcus torques* group after radical surgery.

Surprisingly, our study did not show a notable impact of NAC on gut microbiome composition. These findings contradict several previous studies of various malignancies [25,26,27,28], including gastrointestinal cancers [12,29]. For instance, a recent cross-sectional study by Li et al. reported that the gut microbiome of patients with metastatic or locally advanced esophagogastric or colorectal cancer undergoing chemotherapy differed from that of healthy controls, showing increased richness and compositional shifts in cancer patients [29]. However, this study was not longitudinal and did not include pre- and post-treatment comparisons, unlike our study. The longitudinal study by Kong et al. [12] investigated gut microbiome changes during colorectal cancer treatment with radical surgery and adjuvant capecitabine plus oxaliplatin (CapeOx) chemotherapy and showed that chemotherapy had an impact on the changes in the microbiome’s composition. Several methodological differences between Kong et al.’s [12] study and ours should be considered when interpreting these disparate findings. First, we assessed the effect of NAC on the microbiome before surgery, whereas Kong et al. [12] examined the impact of chemotherapy after colorectal resection. Second, we investigated the effects of FLOT chemotherapy in GC patients, while Kong et al. [12] focused on CapeOx in colorectal cancer patients. Most importantly, the timing of sample collection differed significantly. We collected samples approximately four weeks after NAC, just before surgery, whereas Kong et al. [12] collected samples after each cycle of chemotherapy. This suggests that chemotherapy-induced changes in the gut microbiome may be transient, with a four-week interval possibly allowing the microbiome to recover and reach a stable state. Another study by Chen et al. investigated the impact of surgery and chemotherapy on gut microbiome composition in GC patients [30]. Despite its retrospective cross-sectional design and lack of specificity regarding the type of chemotherapy and surgery, the study found no significant differences in gut microbiome composition between patients treated with and without chemotherapy [30], which aligns with our present findings. To sum up, this longitudinal study is the first to demonstrate that FLOT chemotherapy has no notable and lasting impact on gut microbiome composition in GC patients 4 weeks after the completion of neoadjuvant treatment.

The most important aspect of the present study is the demonstration that, contrary to NAC, radical surgery has a notable and relatively long-lasting impact on gut microbiome composition. Increased oralization-associated bacteria such as *Streptococcus* and *Escherichia-Shigella* genera can characterize radical surgery-induced dysbiosis. Our previous proof-of-concept study proposed that gastrectomy-associated gut microbiome oralization is a significant side effect of the treatment characterized by an increased abundance of bacteria typically found in the oral cavity, including *Escherichia-Shigella*, *Enterococcus*, *Streptococcus*, *Veillonella*, *Oribacterium*, and *Mogibacterium* [6]. The cross-sectional design of the previous study prevented us from drawing irrefutable conclusions. Therefore, this longitudinal study was necessary to confirm the hypothesis that the loss of the gastric barrier following gastrectomy creates conditions for oral cavity bacteria to colonize the distal gastrointestinal tract [6]. Moreover, our previous study demonstrated that gut microbiome oralization is linked to gastrointestinal symptoms, such as bloating, diarrhea, and abdominal discomfort, in GC survivors [6]. This oralization process is mainly associated with intestinal inflammation and the presence of *Streptococcus* in the stool [6]. Chronic intestinal inflammation is a known factor in the pathogenesis of chronic diarrhea [31], a common issue affecting about 40% of long-term survivors after gastrectomy [4,32,33,34]. The present study convincingly confirmed that gastrectomy increases the abundance of Streptococcus, a prevalent bacterial taxon in the oral cavity and commonly implicated in PPI-induced dysbiosis [8,9,35,36,37]. Additionally, the present study showed a radical surgery-induced increase in *Escherichia-Shigella* and lower levels of *Faecalibacterium* and the *Ruminococcus* genera. *Escherichia* is commonly implicated in small intestinal bacterial overgrowth [38], which frequently occurs in patients after gastrectomy and is associated with intestinal and postprandial symptoms [39]. Members of the genus *Escherichia–Shigella* are not sensitive to pH variations in their environment, and they appear to benefit from the altered milieu, as evidenced by their increased abundance observed in the general population after PPI intake [8,40]. In previous reports, *Ruminococcus* species have been associated with a stable human microbiome [41]. Decreased abundance of *Ruminococcus* has been linked to diarrhea in a porcine animal model [42], and specifically, *Ruminococcus* 1 was found to be depleted in GC survivors suffering from diarrhea [6].

It is not surprising that the type of surgery had no significant impact on dysbiosis, as shown in our results. Total gastrectomy leads to an instant loss of the gastric barrier, while subtotal gastrectomy results in a significant increase in gastric pH due to reduced gastric secretion, raising it above the threshold value of 4, which is considered necessary for a potent bactericidal effect [43,44].

To our surprise, the present study failed to show a surgery-induced increase in intestinal inflammation measured by fecal calprotectin. These findings contradict our previous study, which demonstrated elevated fecal calprotectin levels in gastrectomized patients compared to healthy controls [6]. However, the current longitudinal study compared post-surgery calprotectin levels with baseline levels. It is known that fecal calprotectin levels are elevated in patients with esophagogastric cancer [45], which was the case at our baseline timepoint. This pre-existing increase in calprotectin due to cancer may have masked any additional rise induced by the surgery, preventing us from detecting a surgery-induced increase in intestinal inflammation.

This study has some limitations that need to be considered. It is a side analysis of a previous randomized controlled trial [3], where the impact of treatment on gut microbiome composition was a secondary endpoint. Consequently, patients were enrolled without prior statistical power calculations to determine the optimal cohort size. As a result, the cohort size is relatively small, and the findings may require validation in a larger cohort. Also, expanding sample collection across multiple time points after NAC and radical surgery would be beneficial in improving the coherence and persuasiveness of the research findings. This approach would provide a more comprehensive depiction of the dynamic changes in the gut microbiome. However, this study also has some clear benefits. First, it employs a longitudinal design, allowing observation of changes in the microbiome throughout the complete treatment period. Additionally, the sequencing data used are of high quality, enabling precise data analysis that considers even the least common bacteria. Thus, the study provides clear evidence of how the gut microbiome changes throughout GC treatment with NAC and radical surgery.

The findings of the present study have significant clinical relevance. The results demonstrate that radical surgery, rather than NAC, exerts a long-term impact on the gut microbiome, resulting in oralization-defined dysbiosis. When combined with existing evidence associating this specific dysbiosis with gastrointestinal symptoms frequently reported by long-term gastric cancer (GC) survivors, these findings underscore the gut microbiome as a compelling therapeutic target for alleviating such symptoms. Future studies should explore gut microbiome-modulating interventions, such as probiotics, to alleviate gastrointestinal symptoms in long-term GC survivors.

## 5. Conclusions

Radical treatment of advanced GC with NAC and radical surgery has long-term effects on the gut microbiome, which is evident one year after treatment initiation. These sustained alterations primarily stem from radical gastrectomy rather than the NAC.

## Figures and Tables

**Table 1:** Baseline and treatment characteristics of the study patients.

Characteristic		x
Age (years), mean (SD)		60 (11)
Male: Female		24:14
Charlson comorbidity index score, mean (SD)		4 (1.4)
BMI (kg/m^2^), mean (SD)		25 (4)
cT, n (%)	1–2	4 (10.5)
	3–4	34 (89.5)
cN, n (%)	0	10 (26.3)
	1–3	28 (73.7)
cM, n (%)	0	36 (94.7)
	1	2 (5.3)
Tumor localization, n (%)	Upper third	6 (15.8)
	Middle third	14 (36.8)
	Lower third	16 (42.1)
	Total	2 (5.3)
Type of neoadjuvant chemotherapy, n (%)	FLOT	36 (94.7)
	Cisplatin+5-FU	2 (5.3)
Number of cycles, mean (SD)		4 (1)
Type of surgery, n (%)	Total gastrectomy	11 (28.9)
	Subtotal gastrectomy	27 (71.1)
R0, n (%)		37 (97.4)
D2 lymphadenectomy, n (%)		38 (100)
Laparoscopic surgery, n (%)		10 (26.3)
ypT, n (%)	0	2 (5.3)
	1	8 (21.0)
	2	4 (10.5)
	3	17 (44.7)
	4	7 (18.5)
ypN, n (%)	0	16 (42.1)
	1	10 (26.3)
	2	6 (15.8)
	3	6 (15.8)
ypM, n (%)	0	38 (100)
	1	0 (0)

## Data Availability

This side-study of a pilot two-arm randomized clinical trial is registered in the https://clinicaltrials.gov/ (NCT04223401) registry. Raw sequencing data are made publicly available in the National Centre for Biotechnology Information (NCBI) sequence read archive (SRA) at https://www.ncbi.nlm.nih.gov/sra (SRA data accession No. PRJNA1124058). Other data are available from the corresponding author upon reasonable request.

## Supplementary Materials

The following supporting information can be downloaded at: https://www.mdpi.com/article/10.3390/cancers16234074/s1, Table S1: Linear mixed-effects model results for alpha-diversity parameters; Table S2: PERMANOVA analysis results of microbiome composition changes throughout the treatment of gastrointestinal cancer; Table S3: Linear mixed-effects model results for the relative abundance of the most common phylum, when the effect of NAC is compared to the baseline and radical surgery (SX). The table presents the estimates, standard errors (S.E.), t-values (t val.), degrees of freedom (d.f.), and *p*-values; Table S4: Linear mixed-effects model results for the relative abundance of the most common genera, when the effect of NAC is compared to the baseline and radical surgery (SX). The table presents the estimates, standard errors (S.E.), t-values (t val.), degrees of freedom (d.f.), and *p*-values; Table S5: Linear model results for the relative abundance of the most common genera when subtotal gastrectomy is compared to total gastrectomy.
