# Supplementary material for: Gut Microbiome Changes After Neoadjuvant Chemotherapy and Surgery in Patients with Gastric Cancer
Source: Cancers (Basel). 2024 Dec 5;16(23):4074. doi: 10.3390/cancers16234074 (PMC11640656; doi:10.3390/cancers16234074)
Supplement: Supplementary file 1 [file cancers-16-04074-s001.zip › cancers-3343571-supplementary.pdf]

Supplementary information

Gut Microbiome Changes After Neoadjuvant Chemotherapy and Surgery in Patients with Gastric Cancer

Kristina Žukauskaitė[1,2], Bernardas Baušys[1,4], Angela Horvath[2,3], Rasa Sabaliauskaitė[1,5], Agnė Šeštokaitė[1,5], Agata Mlynska[6,7], Sonata Jarmalaitė[1,5], Vanessa Stadlbauer[2,3], Rimantas Baušys[8], Augustinas Baušys[1,8,9]*

[1] Institute of Biosciences, Life Science Center, Vilnius University, Vilnius, Lithuania;

[2]Division for Gastroenterology and Hepatology, Department of Internal Medicine, Medical University of Graz, Graz, Austria;

[3] Center for Biomarker Research in Medicine (CBmed GmbH), Graz, Austria;

[4] Institute of Clinical Medicine, Faculty of Medicine, Vilnius University, Vilnius, Lithuania;

[5] Laboratory of Genetic Diagnostics, National Cancer Institute, Vilnius, Lithuania;

[6] Laboratory of Immunology, National Cancer Institute, Vilnius, Lithuania;

[7] Department of Chemistry and Bioengineering, Vilnius Gediminas Technical University, Vilnius, Lithuania;

[8] Department of General and Abdominal Surgery and Oncology, National Cancer Institute, Vilnius, Lithuania;

[9] Laboratory of Experimental Surgery and Oncology, Translational Health Research Institute, Faculty of Medicine, Vilnius, Lithuania.

Corresponding Author
Augustinas Baušys, MD, PhD
Department of Abdominal Surgery and Oncology
National Cancer Institute, Santariskiu str. 1,
LT-08660, Vilnius, Lithuania;
Phone: +370 6 23 63 865;
E-mail: augustinas.bausys@nvi.lt or augustinas.bausys@gmail.com

Supplementary data

Table S1. Linear mixed-effects model results for alpha-diversity parameters. The table presents the estimates, standard errors (S.E.), t-values (t val.), degrees of freedom (d.f.), and p-values for five alpha diversity indices: Richness, Shannon, Inverse Simpson, and Evenness, and PD—whole tree. It compares baseline and gastrectomy with neoadjuvant chemotherapy treatment.

Source of variation	Est.	S.E.	*t* val.	d.f.	*p*-value
Richness index					
BL	21.408	41.229	0.519	64.068	0.605
post-SX	-206.852	43.085	-4.801	65.468	**<0.0001**
Shannon index					
BL	0.158	0.105	1.504	64.327	0.137
post-SX	-0.278	0.110	-2.524	65.535	**0.014**
Inverse Simpson index					
BL	13.679	10.886	1.257	65.169	0.213
post-SX	-20.512	11.397	-1.800	65.881	0.076
Evenness index					
BL	0.021	0.011	1.837	65.003	0.071
post-SX	-0.009	0.012	-0.791	66.111	0.432
PD – whole tree					
BL	1.828	1.076	1.699	64.891	0.094
post-SX	-4.786	1.126	-4.252	65.923	**<0.0001**

Table S2. PERMANOVA analysis results of microbiome composition changes throughout the treatment of gastrointestinal cancer.

Source of variation	Df	Sum of sq.	R^2	F	*p*-value
**BASELINE *vs.* NAC**					
PCoA based on unifrac					
Treatment Timepoint	1	0.2671	0.01045	0.7182	0.055
PCoA based on wunifrac					
Treatment Timepoint	1	0.000073	0.00786	0.5388	0.537
PCoA based on bray					
Treatment Timepoint	1	0.2226	0.00832	0.5708	0.420
PCoA based on jaccard					
Treatment Timepoint	1	0.3031	0.01005	0.6901	0.375
**NAC *vs.* GASTRECTOMY**					
PCoA based on unifrac					
Treatment Timepoint	1	0.4726	0.02017	1.2761	**0.001**
PCoA based on wunifrac					
Treatment Timepoint	1	0.0002823	0.03065	1.9607	**0.001**
PCoA based on bray					
Treatment Timepoint	1	0.5858	0.02313	1.4678	**0.001**
PCoA based on jaccard					
Treatment Timepoint	1	0.548	0.0195	1.233	**0.001**

Table S3. Linear mixed-effects model results for the relative abundance of the most common phylum, when the effect of NAC is compared to the baseline and radical surgery (SX). The table presents the estimates, standard errors (S.E.), t-values (t val.), degrees of freedom (d.f.), and *p*-values.

Source of variation	Est.	S.E.	*t* val.	d.f.	*p*-value
p_Firmicutes					
BL	-0.036	0.034	-1.068	68.856	0.289
post-SX	-0.157	0.036	-4.407	70.517	**<0.0001**
p_Bacteroidetes					
BL	0.013	0.031	0.431	68.509	0.668
post-SX	0.098	0.033	2.999	69.745	**0.004**
p_Proteobacteria					
BL	0.022	0.015	1.466	99.000	0.146
post-SX	0.072	0.015	4.644	99.000	**<0.0001**
p_Actinobacteria					
BL	-0.001	0.007	-0.193	64.098	0.848
post-SX	-0.023	0.007	-3.382	64.894	**0.001**

Table S4. Linear mixed-effects model results for the relative abundance of the most common genera, when the effect of NAC is compared to the baseline and radical surgery (SX). The table presents the estimates, standard errors (S.E.), t-values (t val.), degrees of freedom (d.f.), and *p*-values.

Source of variation	Est.	S.E.	*t* val.	d.f.	*p*-value
g_Bacteroides					
BL	0.010	0.016	0.593	64.741	0.555
post-SX	0.009	0.017	0.534	65.513	0.595
g_Prevotella 9					
BL	-0.008	0.019	-0.414	67.248	0.680
post-SX	0.047	0.020	2.337	68.025	**0.022**
g_Lactobacillus					
BL	-0.020	0.019	-1.040	61.598	0.302
post-SX	-0.055	0.020	-2.736	63.081	**0.008**
g_Streptococcus					
BL	-0.013	0.018	-0.691	66.891	0.492
post-SX	0.051	0.019	2.642	68.131	**0.010**
g_Holdemanella					
BL	-0.005	0.010	-0.441	67.572	0.661
post-SX	-0.014	0.011	-1.264	68.576	0.211
g_Blautia					
BL	0.000	0.005	0.079	62.209	0.937
post-SX	0.003	0.005	0.555	62.490	0.581
g_Escherichia-Shigella					
BL	0.010	0.011	0.935	99.000	0.352
post-SX	0.037	0.011	3.386	99.000	**0.001**
g_Collinsella					
BL	-0.001	0.006	-0.196	64.197	0.845
post-SX	-0.017	0.006	-2.917	65.065	**0.005**
g_Faecalibacterium					
BL	0.010	0.005	1.948	65.900	0.056
post-SX	-0.012	0.005	-2.214	66.492	**0.030**

g_Roseburia					
BL	-0.007	0.006	-1.141	70.964	0.258
post-SX	0.004	0.006	0.592	72.703	0.555
g_Eubacterium hallii					
BL	0.000	0.004	0.033	67.796	0.974
post-SX	-0.001	0.004	-0.396	68.851	0.693
g_Christensenellaceae R-7 group					
BL	0.010	0.005	2.089	65.425	**0.041**
post-SX	-0.002	0.005	-0.457	65.691	0.649
g_Ruminococcus torques group					
BL	-0.001	0.004	-0.177	61.410	0.860
post-SX	-0.012	0.004	-2.864	62.994	**0.006**
g_Ruminococcaceae UCG-002 group					
BL	0.004	0.003	1.130	64.525	0.263
post-SX	-0.002	0.004	-0.621	65.713	0.537
g_Agathobacter					
BL	-0.005	0.007	-0.691	61.987	0.492
post-SX	-0.008	0.007	-1.180	64.035	0.242

Table S5. Linear model results for the relative abundance of the most common genera when the subtotal gastrectomy is compared to total gastrectomy. The table presents the estimates, standard errors (S.E.), t-values (t val.), and *p*-values.

**Source of variation**	**Est.**	**S.E.**	***t* val.**	***p*-value**
g_Bacteroides				
Total gastrectomy	0.064	0.028	2.260	**0.031**
g_Prevotella_9				
Total gastrectomy	0.070	0.054	1.299	0.204
g_Lactobacillus				
Total gastrectomy	-0.013	0.012	-1.078	0.290
g_Streptococcus				
Total gastrectomy	-0.013	0.059	-0.214	0.832
g_Holdemanella				
Total gastrectomy	-0.022	0.021	-1.078	0.290
g_Blautia				
Total gastrectomy	-0.019	0.013	-1.437	0.161
g_Escherichia_Shigella				
Total gastrectomy	0.007	0.023	0.302	0.765
g_Collinsella				
Total gastrectomy	-0.005	0.005	-0.861	0.396
g_Faecalibacterium				
Total gastrectomy	-0.005	0.005	-0.992	0.329
g_Roseburia				
Total gastrectomy	0.001	0.011	0.093	0.927
g_Eubacterium_hallii				
Total gastrectomy	-0.008	0.007	-1.155	0.257
g_Christensenellaceae_R7_group				
Total gastrectomy	-0.008	0.011	-0.736	0.468
g_Ruminococcus_torques_group				

Total gastrectomy	0.004	0.005	0.803	0.428
g_Ruminococcaceae_UCG_002				
Total gastrectomy	-0.004	0.007	-0.585	0.563
g_Agathobacter				
Total gastrectomy	-0.012	0.010	-1.150	0.259
